# Study protocol for the recreational stimulation for elders as a vehicle to resolve delirium superimposed on dementia (Reserve For DSD) trial

**DOI:** 10.1186/1745-6215-12-119

**Published:** 2011-05-11

**Authors:** Ann M Kolanowski, Donna M Fick, Mark S Litaker, Linda Clare, Doug Leslie, Malaz Boustani

**Affiliations:** 1 School of Nursing, Pennsylvania State University, University Park, PA., USA; 2 School of Dentistry, University of Alabama, Birmingham, AL., USA; 3 Department of Psychology, Bangor University, Wales, UK; 4 Department of Public Health Sciences, Pennsylvania State University, Hershey, PA., USA; 5 Indiana University Center for Aging Research and Regenstrief Institute, Inc, Indianapolis, IN., USA

## Abstract

**Background:**

Delirium is a state of confusion characterized by an acute and fluctuating decline in cognitive functioning. Delirium is common and deadly in older adults with dementia, and is often referred to as delirium superimposed on dementia, or DSD. Interventions that treat DSD are not well-developed because the mechanisms involved in its etiology are not completely understood. We have developed a theory-based intervention for DSD that is derived from the literature on cognitive reserve and based on our prior interdisciplinary work on delirium, recreational activities, and cognitive stimulation in people with dementia. Our preliminary work indicate that use of simple, cognitively stimulating activities may help resolve delirium by helping to focus inattention, the primary neuropsychological deficit in delirium. Our primary aim in this trial is to test the efficacy of Recreational Stimulation for Elders as a Vehicle to resolve DSD (RESERVE- DSD).

**Methods/Design:**

This randomized repeated measures clinical trial will involve participants being recruited and enrolled at the time of admission to post acute care. We will randomize 256 subjects to intervention (RESERVE-DSD) or control (usual care). Intervention subjects will receive 30-minute sessions of tailored cognitively stimulating recreational activities for up to 30 days. We hypothesize that subjects who receive RESERVE-DSD will have: decreased severity and duration of delirium; greater gains in attention, orientation, memory, abstract thinking, and executive functioning; and greater gains in physical function compared to subjects with DSD who receive usual care. We will also evaluate potential moderators of intervention efficacy (lifetime of complex mental activities and APOE status). Our secondary aim is to describe the costs associated with RESERVE-DSD.

**Discussion:**

Our theory-based intervention, which uses simple, inexpensive recreational activities for delivering cognitive stimulation, is innovative because, to our knowledge it has not been tested as a treatment for DSD. This novel intervention for DSD builds on our prior delirium, recreational activity and cognitive stimulation research, and draws support from cognitive reserve theory.

**Trial registration:**

ClinicalTrials.gov identifier: NCT01267682

## Background

Delirium is a state of confusion characterized by an acute and fluctuating decline in cognitive functioning[1]. The exact cause of delirium is unknown but typically involves a vulnerable patient and a noxious insult such as surgery, infection, or adverse effects from medications[2]. Delirium is common and deadly in older adults with dementia, and is often referred to as delirium superimposed on dementia, or DSD. Over 80% of older adults with dementia experience delirium when hospitalized, and studies report that between 24 and 76% die within one year of the index episode [3,4]. Delirium often persists long past the acute phase of an illness and substantially worsens outcomes in a population already burdened with physical and cognitive deficits [5,6]. Over two thirds of older adults admitted to post acute care exhibit delirium on admission[7]. Unresolved delirium results in an accelerated trajectory of cognitive and physical decline that prolongs hospitalization and rehabilitation, and precipitates premature nursing home placement [4,8-10].

It is difficult to prevent delirium in persons who already have reduced cognitive reserve [11]. Efforts to resolve established delirium are critical to implement because data indicate that when delirium resolves slowly, or never at all, less than 50% of pre-illness functioning is realized[12]. These individuals are at high risk for poor quality of life and institutionalization because of their resultant functional impairments, their care becoming too burdensome for family caregivers to support. Interventions that slow the accelerated downward spiral accompanying DSD have the potential to make a major public health impact by preserving function, preventing premature institutionalization and reducing the $152 billion national burden attributed to delirium [4,13,14].

Unfortunately, interventions that treat DSD are not well-developed because the mechanisms involved in its etiology are not completely understood. Historically, the pathophysiology of delirium has been characterized as a derangement of the functional metabolism of the brain[15]. In support of this theory, studies have found decreased blood flow in varied and diffuse regions of the brain that normalizes once the delirium resolves[16,17]. On a cellular level there is disruption of cholinergic transmission[2,18], dopaminergic excess[19], and elevated markers of inflammation (chemokines and cytokines)[20].

The primary neuropsychological deficit in delirium is in the domain of attention; orientation, memory, abstract thinking and executive function are also affected[21]. These deficits are responsible for the cognitive decline seen in delirium, and as improvement in these domains occurs, the fluctuating course typical of delirium resolves[22,23].

Deficits in physical function, i.e., activities of daily living (ADLs) and instrumental activities of daily living (IADLs) parallel the changes in cognition that accompany delirium[4,24-26]. Attentional impairments adversely affect memory performance and may underlie the impaired ADLs and IADLs seen in delirium [27,28]. Attentional impairments affect loco-motor function and increase the risk of falls and accidents [28-30]. Individuals who experience the acute cognitive problems associated with delirium are likely to experience problems with continence, ambulation, dressing, and general way-finding, among other functional tasks.

Very few studies have examined unique risk factors for delirium in persons with dementia, although the available evidence seems to indicate that they are similar to factors observed in cognitively intact individuals. Voyer and colleagues found that advanced age, dementia severity, pain, depression, dehydration, function, behavior, number of medications, and fever were all predisposing risk factors for DSD[31]. In a retrospective chart review of 199 patients with Alzheimer's Disease (AD), urinary tract infection (UTI), surgery, stress/bereavement (death of spouse, change in residence), and severe pain occurred more frequently in delirious than non-delirious patients[32]. Using administrative data, our retrospective review of 7,347 persons with dementia found that those with DSD were older and treated for higher rates of cerebrovascular disease, UTI, dehydration, and pneumonia[33]. Severity of delirium has been linked to later stages of dementia[23] and presence of depression[31].

Certain classes of medications are also strong risk factors for delirium. Persons with dementia take a greater number of central nervous system (CNS)-active medications than their cognitively intact counterparts, including antipsychotics, anxiolytics, and antidepressants[34]. These drugs are known to increase risk of delirium and further cognitive deterioration because of their potent anticholinergic properties [35,36]. Even CNS-active drugs that are appropriately used can accumulate in amounts that lead to delirium, sedation, and falls[37].

Also important for this study are data that indicate a relationship between presence of the apolipoprotein E (ApoE) *E4 allele and delirium severity and duration. Though findings have not always been consistent[38], the weight of the evidence indicates that having at least one copy of the *E4 allele is associated with an increased risk of delirium in young and older adults as well as a more protracted course, independent of demographic and clinical covariates, or premorbid cognitive impairments[39-42].

Clinical management of established delirium is quite variable and includes interventions that target risk factors associated with delirium, but there is no strong empirical basis for prescribing these interventions[43]. The results of clinical trials have been quite modest, and, in some cases, treatments have been ineffective [44-46]. For example, some data indicate that use of antipsychotics and benzodiazepines may actually precipitate delirium and contribute to further long term cognitive impairment[47,48]. Safe, efficacious, and cost-effective non-pharmacological treatments for delirium are urgently needed.

We have developed a theory-based intervention for DSD that is derived from the literature on cognitive reserve and based on our prior interdisciplinary work on delirium, recreational activities, and cognitive stimulation in people with dementia. Cognitive reserve is a construct used to explain the often noted lack of association between clinical manifestations of brain disease and actual brain pathology. It includes both passive and active processes that modify risk for the clinical expression of disease. Passive reserve, sometimes referred to as "brain reserve," is accounted for by brain size and synapse density[49]. It is hypothesized that individuals with larger brains and greater synapse density can tolerate more extensive pathology before they reach the threshold at which symptoms become clinically evident. Active reserve or "cognitive reserve," refers to the efficiency with which an individual can use alternate networks or cognitive strategies to cope with the brain pathology. Cognitive reserve is related to the brains metabolic activity[50] and is dynamic, active, and can be modified by mental activity. Brain reserve and cognitive reserve are not mutually exclusive. Mental activity is a strong signal for the generation of neurons and synapses[51] and, as discussed below, evidence for the ability to develop compensatory mechanisms in late life and early dementia is now emerging.

Epidemiological studies indicate that individual differences in cognitive reserve are due to life-time differences in mental activity; and these activities are considered markers of cognitive reserve. Individuals with more formal education, who are employed in occupations that are characterized by greater complexity and who engage in stimulating leisure activities appear to have greater cognitive reserve than those who participate in fewer mentally stimulating activities as evidenced by their lower risk for dementia and later manifestation of clinical symptoms when a dementia develops [52].

Studies have examined the association of markers of cognitive reserve and incident delirium. They indicate that individuals with low levels of educational attainment and deprivation (living alone) are at greater risk for delirium than individuals with more education or less deprivation[8,14,53]. Because education typically occurs in early life and is not the only source of mental activity over a lifetime, it is important to consider all forms of complex activities when assessing cognitive reserve[54]. A lifetime of low mental activity may lead to low cognitive reserve and increase vulnerability to noxious events that precipitate delirium.

Both delirium and dementia are conditions of reduced cognitive reserve[2,55,56] and have common risk factors: a lifetime of low engagement in complex mental activities[57] and presence of the ApoE *E4 allele[58]. Some investigators have suggested that because delirium and dementia share many clinical, metabolic, and cellular manifestations indicative of reduced cognitive reserve[55,59], they may be part of the same disorder[2,59]. It is plausible, then, that interventions that improve cognitive reserve in dementia may also be effective in delirium.

There is growing evidence that cognitively stimulating activities improve cognitive functioning in healthy older individuals and those in mild to moderate stages of dementia[60-63]. Cognitive stimulation is a specific form of cognitive-focused intervention, defined as a non-regimented intervention that involves engagement in a range of activities that promote cognitive processing aimed at general enhancement of mental and social functioning[64,65]. In this study we provide cognitive stimulation by using recreational activities that are tailored to individual interests and function and that encourage processing in the multiple cognitive domains affected by delirium.

A number of reviews and meta-analyses have assessed the effect of cognitive-focused interventions on cognitive function in older adults with dementia. Unlike in healthy populations, cognitive training has not received compelling support when used in people with dementia[66,67]. However, the benefits of cognitive stimulation for slowing cognitive decline in Mild Cognitive Impairment (MCI) and in mild to moderate stages of dementia are more robust[68-70]. In a trial that used mental status as primary outcome, effect sizes for cognitive gains associated with cognitive stimulation were comparable to those reported for the acetylcholinesterase inhibitors[62]. A recent meta-analysis of cognitive-focused interventions for dementia[71] found that cognitive stimulation had stronger effects for improving cognitive function than cognitive training. Outcome measures varied from study to study but included attention, orientation, memory, abstract thinking and executive functioning. Effects were greater when delivered in individual as opposed to group sessions. There is also some evidence that the effects of cognitive stimulation can be enhanced when combined with cholinergic drugs[72] but these effects deteriorate after one year of treatment[73]. Based on the evidence for the clinical and cost-effectiveness of cognitive stimulation, the National Institute for Health and Clinical Excellence in the U.K. has included this therapy in its guidelines for the non-pharmacological treatment of the cognitive symptoms of dementia[74].

Most of the research on the effects of cognitive stimulation has focused on the progression of dementia. Clinical observations[75] and our preliminary work[76] indicate that use of tailored cognitively stimulating recreational activities may also help resolve DSD. In two clinical trials[77,78] we demonstrated that we can capture and sustain attention in nursing home residents with dementia when we use recreational activities that match individual interests and abilities. Individual interests are important in the design of cognitive activities because when people with dementia are intrinsically motivated to participate in cognitive remediation they obtain greater cognitive and functional benefits[79]. Interesting and enjoyable activities facilitate cognitive processing in the cognitive domains affected by delirium: attention, orientation, memory, abstract thinking, and executive functioning[21]. Cognitive processing helps restore cognitive functioning[64,65], and in persons with delirium, improved cognitive function is accompanied by improvement in physical function[4,24] and resolution of delirium[22,80].

We conducted a pilot study to assess the feasibility of implementing our intervention in a post acute care setting with newly admitted patients who presented with DSD[76]. We provided cognitive stimulation by using recreational activities that were tailored to individual interests and function and that encouraged processing in the multiple cognitive domains affected by delirium. All participants had a diagnosis of dementia on admission and at least 2 symptoms of delirium as assessed by the Confusion Assessment Method [105]. We randomized 16 subjects to treatment with cognitively stimulating activities (N = 11) or usual care control (N = 5) and followed participants for 30 days. Blinded research assistants conducted daily assessments of delirium, delirium severity and functional status. Participants in the treatment group received activities for 30 minutes each day. Analyses indicated that the control group had a significantly greater decrease in physical function and mental status over time compared to intervention. Severity of delirium approached significance and improvement over time favored intervention. Although not statistically significant, there was a difference in mean (7.0 vs. 3.27) and median (7.0 vs. 3.0) days with delirium: the control group having more days of delirium. We were encouraged by these results and are ready to conduct a randomized clinical trial of treatment efficacy.

**Figure 1:**
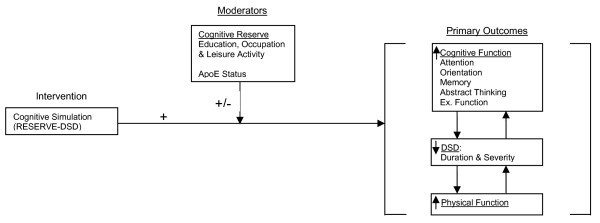
**Hypothesized Model**.

This paper describes the study protocol for our randomized clinical trial (RCT), referred to as Recreational Stimulation for Elders as a Vehicle to resolve DSD (RESERVE- DSD) in which we test these cognitively stimulating activities. The aims of the trial are: Aim 1) to demonstrate the efficacy of the RESERVE-DSD intervention for resolving delirium; Aim 2) to evaluate potential moderators (markers of cognitive reserve) of RESERVE-DSD efficacy; and secondary Aim 3) to describe the costs associated with implementing the intervention. Our hypothesized model of intervention effect is diagramed in Figure 1.

## Methods/Design

**Figure 2:**
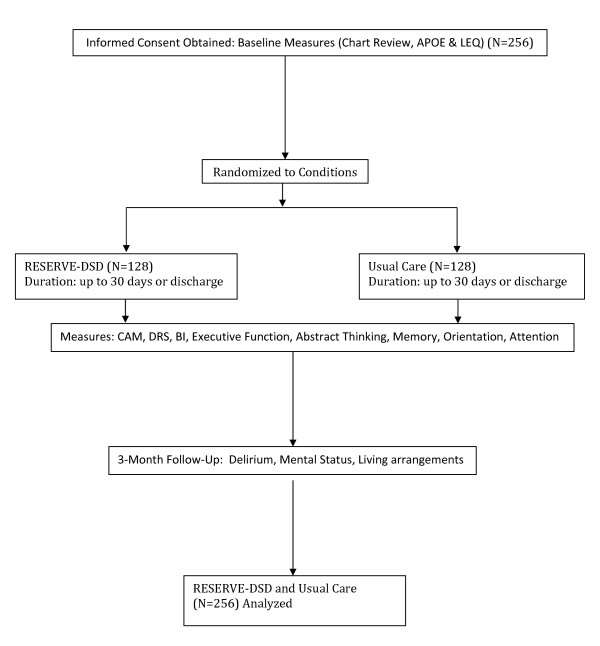
**Reserve-DSD Trial Flow Diagram**.

This randomized repeated measures clinical trial will involve participants being recruited and enrolled at the time of admission to post acute care, and then randomly assigned to one of two conditions: RESERVE-DSD (intervention) or usual care (control) (See Figure 2: Trial flow diagram.)The study protocol was approved by the Penn State Institutional Review Board (IRB# 33443). Participants in the intervention group will receive nursing care that is routinely delivered for their medical/surgical condition including participation in their prescribed therapies plus RESERVE-DSD. At the present time there is no standard care for DSD[43,81]. Participants in the usual care group will receive nursing care that is routinely delivered for their medical/surgical condition including participation in their prescribed therapies.

The two-group experimental design we propose may not completely control for the many variables that can affect DSD. We will control as much as possible for factors we believe are most likely to impact our findings through strict enrollment criteria, and by considering several potential covariates in our analyses: dementia stage, co-morbidities, age, use of CNS-active drugs, pain, infection, dehydration, APOE status, and lifetime complex mental activity including years of formal education, occupation, and past leisure activity.

### Setting and Participants

The study will be conducted in five Northeast, Central, and South-central Pennsylvania nursing homes that provide rehabilitation services (post acute/skilled nursing care). Sites were selected to ensure a large population for recruitment and to increase the diversity of the sample. To improve the potential for translation into practice and to increase the generalizability of our findings we have selected a mix of for-profit, nonprofit, county-owned, rural and urban, and large, medium, and small community-based settings.

Participants are individuals with DSD who are admitted to post-acute care following hospitalization. These individuals will meet the following inclusion criteria: age 65 years or older; English speaking; community residing prior to hospitalization; diagnosis of mild to moderate stage of dementia as confirmed by medical chart review and/or a score of 0.5 to 2.0 on the Clinical Dementia Rating Scale[82]and a score of 3 or greater on the Modified Blessed Dementia Rating Scale[83]; and at least two features of delirium as assessed by the Confusion Assessment Method. A consensus panel of three members with expertise in geriatric medicine, neuropsychology, and delirium will adjudicate all dementia and delirium diagnoses. Exclusion criteria include: severe hearing, speech or vision impairments; medical diagnoses of acute major depression, acute psychiatric condition, acute stroke, Parkinson's disease, Huntington's disease, normal pressure hydrocephalus, seizure disorder, subdural hematoma, head trauma, or known structural brain abnormalities; or a life expectancy of less than 6 months. Participants who satisfy all enrollment criteria will be invited to participate in the study.

### Consent Process

Participants in this study will be frail older adults who lack decisional capacity due to their delirium superimposed on dementia. Participants and their legally authorized representative (LAR) will be approached for screening and consent (for those found eligible) at the point of admission to post-acute care. The LAR will sign for the participant and a copy of the consent will be given to them. We ask the participant for assent on a daily basis prior to assessment and intervention.

### Randomization and Control of Cross-Contamination

Randomization will be concealed until after the initial screen and consent are obtained and an ID number is assigned to the participant. The statistician will generate the randomization sequence using a random number generator. Randomization will be conducted in blocks by nursing home site and time to ensure equal assignment across the two groups at the completion of the study and approximately equal assignments throughout the study to control for unknown temporal effects. The statistician will be blinded to treatment coding.

Our team discussed several randomization procedures and we have elected to randomize by participant rather than by site, even though the latter approach is an excellent method for controlling cross-contamination of conditions. There are several reasons for our decision. First, nursing homes are unstable environments[84] and there is evidence indicating that nursing home quality indicators are unstable from one six-month period to the next[85]. Changes in quality indicators may reflect change in quality of care and/or resident profile. Either could potentially impact our outcomes because of their likely effect on usual care. Second, we are confident that we can control treatment contamination within sites, and for the same sample size, randomization by participant will provide greater power than randomization by nursing home.

We control for cross-contamination by conducting all interventions using our trained research assistants (RAs) (not nursing home staff) in an area specifically set aside for these sessions. Nursing homes have provided us with this research space in the past and staff has always respected our procedures for control of cross-contamination. Only the resident scheduled for a particular session will be allowed access to the area for that time period.

### Intervention

Participants in the intervention group will receive nursing care that is routinely delivered for their medical/surgical condition including participation in their prescribed therapies plus RESERVE-DSD. This intervention consists of increasingly challenging recreational activities that are cognitively stimulating and tailored to each participant's interests and functional abilities. The recreational activities target cognitive functions affected by delirium: attention, orientation, memory, abstract thinking, and executive functioning. Participants will receive up to 30 minutes of their respective recreational activities once a day between the hours of 1 pm and 5 pm for up to 30 consecutive days beginning within 24 hours of admission to post acute care.

The cognitively stimulating recreational activities to be studied are from previously designed, well-established programs of therapeutic activities from Drs. Kolanowski and Clare's research on the behavioral and cognitive symptoms of dementia, respectively. We have constructed a large base of cognitively stimulating recreational activities, many taken from the growing literature on brain fitness[75,86-90]. The activities, such as word search, finish the phrase, and name that tune, offer stimulation in multiple cognitive domains, combined in novel ways, as opposed to stimulation in a single cognitive domain like memory training.The investigators have classified the base of recreational activities by most prominent domain stimulated, however, in actuality activities stimulate several cognitive domains simultaneously. For example, the game of "Name that Tune" requires processing in the areas of both attention and memory. Multi-domain cognitive activities demonstrate more robust results than single domain training[91]. The activities are implemented using inexpensive items and are readily available to most nursing homes. Because they require no special expertise to implement, they are well-suited to the resource-stressed nursing home environment.

#### Assessment and Prescription

The selection of activities for each participant will be determined by Drs. Kolanowski and Clare. Each participant's baseline data, including dementia stage, physical function, and activity interests are reviewed. Based on this assessment, three recreational activities tailored to the participant's functional abilities and designed around themes of their interests will be selected by level of difficulty (easy, moderate, and hard) in each cognitive domain. For example, a participant who has arthritic problems with their hands, a mild stage of dementia, and a history of interest in gardening might be prescribed: identify the sound of a lawn mower (attention); discuss whether the current calendar month is good for planting (orientation); memory tray with three or more garden tools (memory); describe steps to planting a garden (abstract thinking); and having the participant plant flower seeds using adapted hand tools (executive functioning). Using the domain of attention as an example, increasing the level of difficulty might proceed in this fashion: lawn mower sound identification (easy); circle the garden tools in a picture (moderate); item search of vegetables embedded in a "busy" picture (difficult). We provide variety from day to day to encourage cognitive processing and to prevent a practice effect.

#### Procedure for Implementation

To encourage initial engagement in the activity, the research assistant (RA) will use the System of Least Restrictive Prompts[92] beginning with verbal cueing, followed by verbal cueing and demonstration of the activity. Participants are then encouraged to read out loud, point out facts, work puzzles, ask questions, answer questions, or make choices. RAs will use principles for implementing cognitive activities outlined by Green & Bavelier[93]: active engagement, incremental increases in task difficulty; verbal encouragement and motivation throughout the session; feedback and praise; and variability in tasks. These approaches maximize cognitive processing and restoration.

#### Scheduling and Duration of Intervention

Participants will receive the intervention for 30 minutes each day for up to 30 consecutive days between the hours of 1 pm and 5 pm. This dosage is based on a consensus report of activity effectiveness compiled by expert recreational therapists from the American Therapeutic Recreation Association and the National Recreation Society[94], standard recreational therapy practice in the nursing home, and studies that have demonstrated the efficacy of daily, 30-minute recreational therapy for behavioral symptoms and functional impairment in persons with dementia[95-97].

#### Treatment Fidelity

We use several methods to monitor and enhance the reliability of our intervention[98,99]. Intervention RAs are trained in a two-day program that we have developed, standardized, and used for many years to prepare RAs for implementing recreational activities with people who have dementia. These RAs need no special educational background because the activities we use are simple, non-technical games, and exercises. RAs will practice in simulated situations until they achieve 100% agreement on all critical elements of activity intervention before going into the field. The project director (PD) will monitor delivery of the treatment by observing each RA conduct interventions on 10% of sessions in the field (randomly selected). Retraining will be initiated if treatment fidelity is not obtained, as assessed by any "no" answer on the treatment fidelity checklist. Additionally, RAs will complete a treatment fidelity check for any intervention session that was not delivered according to protocol. We will monitor receipt and enactment of treatment using measures for participant adherence to the protocol: intervention dose (time on task and level of participation) and duration (number of treatment days received). Our methods and measures will help us to monitor and ensure the reliability of our intervention, its delivery, receipt and enactment.

### Description of Usual Care (Control)

At the present time there is no standard care for DSD [43,81]. Participants in the usual care group will receive nursing care that is routinely delivered for their medical/surgical condition including participation in their prescribed therapies. To describe what constitutes usual care (control) we will conduct medical chart reviews on all participants (intervention and control) and extract data on the following: attendance at prescribed therapies and activity programs; CNS-active drug use (regular and prn); and nursing interventions recorded for behaviors nurses use to describe delirium[100]: confusion, disorientation, altered mental status, agitation, inappropriate behavior, mental status change, inattention, hallucination, and lethargy. Because the nursing home environment is unstable[84,85], we will also record data every six months on staffing ratios at each site, as published on the Pennsylvania Department of Health website, and assess staff knowledge of delirium using our previously developed case vignettes[101]. We will ask all consenting staff (RN, LPN, and CNAs) to complete the vignettes at baseline and then every six months during data collection to capture any change in overall level of delirium knowledge per site that might occur due to historical factors or staff turnover in the nursing home. We will use these data to quantify usual care and to monitor any qualitative differences between sites.

### Measures

#### Baseline Measures

Following consent, the PD will collect baseline data on all participants. From the medical chart the PD will extract demographic data, all medical diagnoses, and all medications and therapies prescribed using an investigator-developed *Baseline Medical Chart Review Form*. For all chart reviews we will incorporate methods that help improve the precision of data abstraction[102,103].

To determine ApoE status, the PD will obtain buccal swabs from all participants using a procedure established by the Huck Institutes of Life Sciences Genome Core Facility (GCF) at Penn State. ApoE status will be determined by extracting DNA from the buccal swabs using a protocol optimized by the Institute of Psychiatry in London[104]. To identify the six ApoE genotypes comprising the ApoE *E2, *E3 and *E4 alleles, two single nucleotide polymorphisms (SNPs) will be assayed using the TaqMan Allele Discrimination method. Based on the distribution in the AD population, we expect that approximately 40-45% of our sample will carry at least one *E4 allele[105].

Assessment of participants' lifetime of complex mental activities, a measure of cognitive reserve, will be obtained by interviewing a knowledgeable informant using the *Lifetime of Experiences Questionnaire (LEQ*)[54] a reliable and valid instrument for assessing educational, occupational, and leisure lifestyle activities that are protective against cognitive decline. The LEQ consists of 42 items constructed around two dimensions: three life stages (young, mid, and late adulthood) and specific vs. non-specific mental activity in each stage. Scores are calculated for each stage and then summed for a total LEQ score. Higher scores indicate higher lifetime mental activity. The LEQ has an overall internal consistency of .66, test-retest reliability of .98 and is discriminate between older adults with high and low mental activity levels. Healthy older adults with higher LEQ scores have shown less cognitive decline over 18 months than those with low scores, independent of covariates[54].

Activity interests will be assessed for participants randomized to RESERVE-DSD by interviewing the responsible party using an adapted version of the *Farrington Leisure History Checklist*[88]. This checklist contains 150 recreational activities categorized by games, social, outdoor and cultural activities. These data will be used to adapt cognitively stimulating recreational activities around themes that match participants' interests.

#### Measures of Primary Outcomes

Our primary outcomes are: *1) delirium *(duration and severity), 2) *cognitive function*, and 3) *physical function*. Because delirium status fluctuates, and because cognitive and physical function also fluctuates with delirium status, we will take daily measures of these outcomes to capture the variability typical of DSD. Blinded RAs will collect outcome data. We will have two separate groups of RAs: one to implement the intervention and the other to conduct daily assessments. Daily assessments will be completed during the morning hours (9 am to 11 am) and the intervention will be implemented during the afternoon hours (1 pm to 5 pm) to ensure that there is no contact between the two groups of RAs

*Delirium *will be measured by a structured interview consisting of questions from the *Montreal Cognitive Assessment *(MoCA)[106] observation and the *Confusion Assessment Method *(CAM)[107]. The MoCA is a brief cognitive screening tool that demonstrates excellent sensitivity (90% and 100%) in MCI and early AD respectively, and specificity (87%). Content validity was established by the high correlation (.87) between the MoCA and MMSE. MoCA items on orientation, memory and language are used in the assessment of delirium. The CAM is a standardized screening algorithm allowing persons without formal training to quickly and accurately identify delirium. The CAM has four features: 1) acute onset and fluctuating course, 2) inattention, and either 3) disorganized thinking, or 4) altered level of consciousness[107]. A participant is scored as having subsyndromal delirium if they exhibit any two features and full delirium if they exhibit features one and two and either three or four[108]. The CAM was validated against geriatric psychiatrists' ratings using DSM-III-R criteria and has been shown to have a sensitivity between 94% and 100% and a specificity between 90% and 95%[107,109]. The CAM has also been validated in persons with dementia. Studies have shown the utility of a daily CAM in identifying delirium and its waxing and waning states[110]. Delirium will be recorded daily by RAs as present or absent; full or subsyndromal; hyperactive, hypoactive or mixed[111]. *Delirium Duration *will be calculated as total number of days with full or subsyndromal delirium. *Delirium severity *will be measured daily using the *Delirium Rating Scale *(DRS)[112],a 16-item clinician-rated scale validated in both delirium and dementia groups, and having good sensitivity, specificity and high interrater reliability (ICC 0.97). Scores range from 0 to 39; higher scores indicate greater severity.

*Cognitive Function *will be measured with several instruments that capture the cognitive domains affected by delirium: attention, orientation, memory, abstract thinking, and executive function. These domains are also affected in dementia, but previous studies have shown that as they improve, DSD resolves[22,80]. *Digit Span *(forward and backward) is a subtest of the Wechsler Adult Intelligence Scale (WAIS) and a classic measure of attention[113]. Participants are given increasingly longer sequences of digits to repeat initially forwards then backwards and receive a point for each correct sequence. The assessment ends when the participant misses two sequences in a row. The maximum possible score is 16 (forward) and 14 (backward). Higher scores indicate better attention and working memory. Median reliabilities reach .97 and .96 for forward and backward spans respectively[114,115]. *MoCA *items on orientation and memory will be used to measure those domains. The *Similarities test *of the verbal Wechsler Adult Intelligence Scales (WAIS) will be used to measure abstract thinking. The *Similarities test *has 18 items requiring the participant to describe how two given things are alike (ex., a hamburger and pizza). The Similarities test measures concrete, functional, and abstract thinking. The WAIS was revised to cover normative data for individuals 16-89 years old with strong reliability and validity reported. Reliability coefficients for the WAIS and *Similarities test *range from .93-.70 and test-retest reliability has been consistently in the .80s[116]. The *CLOX*[117] will be used to measure executive function. It is an easy to administer clock drawing task that elicits impairment in executive function and discriminates it from non-executive constructional failure. The *CLOX *has two parts: *CLOX 1*, a free drawing of a specified time, and CLOX 2, a simple copying task. Both steps are rated on 14 items with scores ranging from 0 to 15, higher scores indicate better executive function. The *CLOX *has an internal consistency of .82, interrater reliability of .94 (CLOX 1) and .93 (*CLOX 2*) and correlates strongly with measures of cognitive function in healthy and cognitively impaired older adults (MMSE and EXIT25).

*Physical Function *will be measured using the *Barthel Index *(BI)[118,119], a commonly used ordinal scale for assessing activities of daily living in patients receiving inpatient rehabilitation. The BI has ten items (seven for self-care and three for mobility) that are scored in steps of five points with a total score range of zero (totally dependent) to 100 (fully independent). The BI is a reliable indicator of functional ability in older adults when administered by face-to-face interview (ICC 0.89) and on testing by different observers (ICC 0.95-0.97)[118,120].

#### Measures of Moderators of RESERVE-DSD

At baseline we will collect data on potential moderators of the intervention: we will use the *Lifetime of Experiences Questionnaire (LEQ)*[54] to obtain a measure of baseline cognitive reserve; and we will obtain *buccal swabs *for DNA extraction to identify the six ApoE genotypes comprising the ApoE *E2, *E3 and *E4 alleles[104]. These measures are described under baseline measures.

#### Measures of Variables Affecting Vulnerability

Dementia stage, age, comorbidity, pain, infection, dehydration and CNS-active drugs. Measures of these variables will be obtained during screening (dementia stage: Clinical Dementia Rating Scale), following consent at baseline (age, comorbidities: baseline chart review), daily (presence of pain), and weekly (infection, dehydration and CNS-active drugs: weekly chart review). We will use the *Charlson Co-morbidity Index*, a weighted index that takes into account the number and seriousness of co-morbid diseases, to calculate a co-morbidity score for each participant[121]. Pain will be measured daily using the Pain Assessment in Advanced Dementia *(PAINAD*) scale which is an observational scale of five items (breathing, vocalization, facial expression, body language, and consolability). We use an observational rather than a verbal scale for greater reliability on days when participants experience greater severity of delirium. *PAINAD *is scored from 0-10 and has an internal consistency reliability of 0.50-0.65 and interrater reliability of 0.82-0.97[122,123]. Infection and dehydration will be identified as present or absent (yes/no) during the weekly chart review. The trained RA will review new medical orders, lab values and nurses notes for indicators of infection and dehydration. CNS-active drugs can further cognitive deterioration because of their potent anticholinergic properties[35,36]. We identify CNS-active drugs administered through a weekly chart review using the American Hospital Formulary Service drug classification[36]. We then calculate the anticholinergic burden associated with these drugs using the *Anticholinergic Cognitive Burden *(ACB) Scale[124,125]. The ACB is an expert based practical index that classifies the severity of a drug's anticholinergic activity on cognition using a scale of 1 (mild), 2 (moderate) and 3 (severe). Total ACB is calculated by summing the ACB scores of all regularly and prn scheduled drugs administered to the participant for that week. We will weigh the ACB score for each drug by days administered during that week before summing for the total ACB score.

#### Measures of Dosage and Satisfaction with Intervention

Intervention efficacy is often related to dose. During each intervention session, dosage will be obtained by the RA using a stop watch to time the minutes and seconds that the participant engaged in activities (*time on task*: 0 to 30 minutes). The RA also rates the *Level of Participation *using a scale developed by Kovatch and Magliocco for measuring extent of participation in recreational activities (an indicator of cognitive processing)[126]. Scores range from 0 to 3 with descriptors for each numerical rating (dozing, null, passive, active), higher scores indicate greater participation. In our work we achieved interrater reliabilities (ICC) of .99 for time on task and .83 for Level of Participation. Total dosage received will be calculated by weighing each daily time on task score by level of participation score and then summing across intervention days. We will use these data to conduct analyses of the effect of dosage on primary outcomes of the intervention. Activities attempted per cognitive domain are recorded daily.

Because we wish to improve the potential for translating our intervention into practice, we will use investigator-developed *Satisfaction Surveys *to assess staff, family, and participant reports of satisfaction with RESERVE-DSD. These brief surveys are collected at the completion of the 30-day intervention period or at discharge, whichever comes first. We will use these data to refine our intervention for a future effectiveness study.

#### Other Measures

Length of stay, discharge disposition, rehospitalization, institutionalization, and death will be obtained by using an investigator-developed *Weekly Chart Review*. To further help describe usual care we will assess staff knowledge of delirium using our case vignettes[101] at baseline and every 6 months to capture any change in overall level of delirium knowledge per site that might occur due to historical factors or staff turnover in the nursing home. The case vignettes include five standardized cases that depict different hospitalized patients experiencing dementia, hypoactive delirium, hyperactive delirium, hyperactive DSD and hypoactive DSD. The case vignettes were intended to assess staff ability to identify different subtypes of delirium and delirium superimposed on dementia in a standardized format, as well as to gather qualitative data from the staff. All case vignettes were completed by 4 expert panelists. Their overall agreement on the cases was 84%, with a kappa of 0.69. For identification of delirium motoric subtype expert agreement was 100% with a kappa of 1.0[101]. We will also record staffing ratios at each site every six months by accessing the Pennsylvania Department of Health website where these data are published.

We will conduct a three-month follow-up to track the trajectory of delirium resolution. We are not powered to conduct analyses with the data from this three-month follow-up. These data will be used to help calculate the sample size needed for a future multisite study that will focus on long term outcomes of RESERVE-DSD effectiveness. The three month point approximates a Medicare benefit period for skilled care (100 days), so we should be able to capture data reflecting variability on these longer term outcomes as participants will likely be either discharged to home, assisted living or transferred to a long-term care facility for continuing care at that point. We will contact the responsible party by phone and obtain information on the participant's length of stay in post acute care (if we do not have this information at the 30 day point), any additional re-hospitalizations, institutionalization and death. We will interview the responsible party using the CAM and telephone MMSE to assess the participant's current delirium status. In a study of 41 older adults discharged to home following hip surgery, the telephone method of assessing delirium showed a sensitivity of 1.00 and specificity of 0.94[127].

### Statistical Analysis

Prior to conducting any statistical analyses, the distributions of all sample data will be examined. Extreme values will be compared with the original data collection forms in order to identify and correct data entry errors. Following this data validation step, sample distributions will be evaluated to determine if they meet the assumptions necessary for use of normal distribution-based statistical methods. If the data are not normally distributed, suitable transformations will be sought to normalize them. Possible transformations include log, square root, and rank transformations. If the data are not sufficiently normalized by transformation, the analysis may proceed using permutation testing to determine significance levels. Categorical variables will be evaluated using models based on the binomial or multinomial distributions. The design of this study is a two-group randomized design within blocks defined by individual nursing homes. Randomization will be conducted at the participant level, so that intervention groups are crossed with nursing homes, rather than nested within them. Repeated measurements will be made within individual participants for up to 30 days. The statistical methods must appropriately account for within-participant correlation among these repeated measurements.

The primary statistical model will be mixed-model analysis of variance (ANOVA). A term representing individual participants will be included as a random effect in the model in order to account for correlated multiple measurements for each participant. Treatment and covariates will be included as fixed effects. The primary outcome variables are delirium severity and duration, and cognitive and physical functioning.

The primary analyses will be based on intent to treat. Thus, participants will be included in the analyses regardless of their level of participation in the treatments or of their loss to follow-up. Participants who have missing data for some time points will not be excluded from analyses. The methodology incorporates all available measurements for each participant into the analysis. Missing values for some observation times will not cause the participant's entire record to be excluded from the analyses, as may occur in usual repeated measures models.

Potential confounders will be included as covariates in the analysis. If a variable substantially alters either the significance level of a group comparison or a measure of association, that variable will remain in the model in order to control for its confounding effect. Variables that will be evaluated as covariates include those that increase delirium severity and duration, acetylcholinesterase inhibitors, and potential moderators of the association between treatment and outcome.

In order to evaluate differences in change across the study period between the intervention groups, time, and group by time interaction terms will be included in the model. A significant group by time interaction will be of interest, as it would reflect differences in rates of change between the groups. A significant interaction term could yield a non-significant test for the main effect of group, but could reflect an important effect of the intervention. If the test for interaction is significant, the nature of the interaction effect will be evaluated using means plots and post-hoc contrasts.

Hypothesized moderator variables will be evaluated using LISREL and SAS software. Structural models will be specified based on the path diagram presented previously. The path diagram will be used to define the hypothesized structure of the relationships among the variables. Multilevel structural equations modeling (SEM) will be used to evaluate the hypothesized structure, including testing the hypothesized moderator status of the cognitive reserve variables. Tests for moderation are based on regression relationships among the specified variables. These relationships are specified in the SEM model and may be tested based on statistical significance of parameters for direct and indirect effects in the hypothesized model. These relationships can also be tested using sets of mixed-model regression analyses[128-130]. This approach, based on the "Baron and Kenny steps"[128] will be implemented as supplemental analyses using SAS software. Using the regression approach, moderating effects are evaluated as the interaction effect of X and M. Least-squares means with 95% confidence intervals will be used to summarize moderated effects.

One goal of the study is to describe the costs associated with the RESERVE-DSD intervention and developing methods for documenting these costs. This information is important for evaluating the public health impact of the intervention. Because this is a new intervention with little data describing its cost, activity-based costing methods will be used. Determining the cost of the intervention will involve several steps. First, resources used to deliver the intervention will be determined. These resources will include personnel, supplies and other expenses. Next, the amounts used of these resources will be documented. In the case of personnel time, the amount of time spent on activities related to the intervention will be determined using activity diaries for assessment and prescription time, and direct observation by study personnel using the *time on task *measure for the intervention. Finally, the amounts used of each resource will be multiplied by the unit cost of each resource to determine the cost associated with the resource. For personnel costs, the time spent by each provider on activities related to the intervention will be multiplied by the hourly compensation, including benefits, associated with the provider. Finally, costs will be summed across all components of the intervention and divided by the number of patients treated to determine the average cost per patient of delivering the RESERVE-DSD intervention. Because different nursing homes might use different types of personnel for some intervention activities, sensitivity analyses will be used to determine the effect of using different types of personnel on the cost of the intervention. Hence, in addition to the actual average cost of providing the intervention, projected costs based on different types of appropriate personnel providing the intervention will also be constructed. These data will inform a future cost-effectiveness study.

## Discussion

Our work and that of others has shown that delirium is a common, deadly, persistent, and costly problem in people with dementia. There is strong support for targeting patients with DSD at admission to post acute care as a high risk group for poor health outcomes. In these settings persistent cognitive decline is a predictor of functional dependency[25]. When delirium is unresolved, rehabilitation is hampered because the associated cognitive problems (i.e., inattention, disorientation etc) interfere with patients' ability to fully engage in restorative therapies. Our goal is to facilitate maximal rehabilitation benefits so these individuals can return to their homes.

The data also show a compelling need to develop non-pharmacological interventions that resolve DSD in a population that is vulnerable to the effects of current pharmacological treatments. Our study is significant because it will address this critically important clinical problem and its lack of effective treatment. We will test the efficacy of cognitive stimulation (RESERVE-DSD), an intervention that has helped improve cognitive functioning in people with dementia, and holds promise for successfully managing those with DSD. This study will also examine important moderators of intervention effectiveness to help us determine those most responsive to treatment. Armed with this knowledge we will be able to tailor RESERVE-DSD with more precision and for greater effect.

RESERVE-DSD has the potential to reduce poor health outcomes that are major sources of today's spiraling health care costs. The societal implications of helping older individuals with dementia regain adequate function after hospitalization in order to return to their homes are enormous in terms of aging in place, quality of life, cost, and caregiver burden. Our theory-based intervention, which uses simple, inexpensive recreational activities for delivering cognitive stimulation, is innovative because, to our knowledge it has not been tested as a treatment for DSD. This novel intervention for DSD builds on our prior delirium, recreational activity and cognitive stimulation research, and draws support from cognitive reserve theory, one of the most exciting perspectives in cognitive research today.

## Abbreviations Used

DSD: Delirium Superimposed on Dementia; Reserve For DSD: Recreational Stimulation for Elders as a Vehicle to resolve DSD;

## Competing interests

The authors declare that they have no competing interests.

## Authors' contributions

AMK and DMF obtained funding for the study. All authors contributed to the design of the study. MSL conceptualized the statistical analysis: LC and AMK designed the intervention; DLL and MSL conceptualized the cost analysis; MB developed the method for adjudicating dementia and delirium diagnoses and the Anticholinergic Cognitive Burden Scale. AMK wrote the first draft of the manuscript. All authors contributed to the next versions of the manuscript and have read and approved the final version.

## References

[B1] American Psychiatric Association Diagnostic and Statistical Manual of Mental Disorders (DSM-IV) 1994 4 Washington, DC: American Psychiatric Press Inc

[B2] Inouye SK Delirium in older persons N Engl J Med 2006 354 1157 1165 10.1056/NEJMra052321 16540616

[B3] Fick DM Agostini JV Inouye SK Delirium superimposed on dementia: A systematic review J Am Geriatr Soc 2002 50 1723 1732 10.1046/j.1532-5415.2002.50468.x 12366629

[B4] McCusker J Cole M Dendukuri N Delirium in older medical inpatients & subsequent cognitive & functional status: A prospective study CMAJ 2001 165 575 583 11563209 PMC81415

[B5] Inouye SK Schlesinger MJ Lydon TJ Delirium: a symptom of how hospital care is failing older persons and a window to improve quality of hospital care Am J Med 1999 106 565 573 10.1016/S0002-9343(99)00070-4 10335730

[B6] McCusker J Cole M Dendukuri N Han L Belzile E The course of delirium in older medical inpatients: a prospective study J Gen Intern Med 2003 18 696 704 10.1046/j.1525-1497.2003.20602.x 12950477 PMC1494920

[B7] Kiely D Bergmann M Murphy K Jones R Orav E Marcantonio E Delirium among newly admitted postacute facility patients: prevalence, symptoms, and severity The Journals of Gerontology Series A: Biological Sciences and Medical Sciences 2003 58 M441 445 10.1093/gerona/58.5.m441 12730254

[B8] Fong T Jones R Shi P Marcantonio E Yap L Rudolph J Yang F Kiely D Inouye S Delirium accelerates cognitive decline in Alzheimer disease Neurology 2009 72 1570 10.1212/WNL.0b013e3181a4129a 19414723 PMC2677515

[B9] Pitkala KH Laurila JV Strandberg TE Tilvis RS Prognostic significance of delirium in frail older people Dement Geriatr Cogn Disord 2005 19 158 163 10.1159/000082888 15627764

[B10] McCusker J Cole M Abrahamowicz M Primeau F Belzile E Delirium predicts 12-month mortality Arch Intern Med 2002 162 457 463 10.1001/archinte.162.4.457 11863480

[B11] Marcantonio ER Flacker JM Wright RJ Resnick NM Reducing delirium after hip fracture: a randomized trial J Am Geriatr Soc 2001 49 516 522 10.1046/j.1532-5415.2001.49108.x 11380742

[B12] Kiely D Jones R Bergmann M Murphy K Orav E Marcantonio E Association between delirium resolution and functional recovery among newly admitted postacute facility patients The Journals of Gerontology: Series A 2006 61 204 208 10.1093/gerona/61.2.204 16510867

[B13] Leslie DL Marcantonio ER Zhang Y Leo-Summers L Inouye SK One-year health care costs associated with delirium in the elderly population Arch Intern Med 2008 168 27 32 10.1001/archinternmed.2007.4 18195192 PMC4559525

[B14] Pompei P Foreman M Rudberg MA Inouye SK Braund V Cassel CK Delirium in hospitalized older persons: outcomes and predictors J Am Geriatr Soc 1994 42 809 815 8046190 10.1111/j.1532-5415.1994.tb06551.x

[B15] Engel GL Romano J Delirium, a syndrome of cerebral insufficiency J Chronic Dis 1959 9 260 277 10.1016/0021-9681(59)90165-1 13631039

[B16] Alsop DC Fearing MA Johnson K Sperling R Fong TG Inouye SK The role of neuroimaging in elucidating delirium pathophysiology J Gerontol A Biol Sci Med Sci 2006 61 1287 1293 17234822 10.1093/gerona/61.12.1287

[B17] Yokota H Ogawa S Kurokawa A Yamamoto Y Regional cerebral blood flow in delirium patients Psychiatry Clin Neurosci 2003 57 337 339 10.1046/j.1440-1819.2003.01126.x 12753576

[B18] Tune LE Damlouji NF Holland A Gardner TJ Folstein MF Coyle JT Association of postoperative delirium with raised serum levels of anticholinergic drugs Lancet 1981 2 651 653 6116042 10.1016/s0140-6736(81)90994-6

[B19] Trzepacz PT Is there a final common neural pathway in delirium? Focus on acetylcholine and dopamine Semin Clin Neuropsychiatry 2000 5 132 148 10837102 10.153/SCNP00500132

[B20] de Rooij SE van Munster BC Korevaar JC Levi M Cytokines and acute phase response in delirium J Psychosom Res 2007 62 521 525 10.1016/j.jpsychores.2006.11.013 17467406

[B21] Wacker P Nunes P Cabrita H Forlenza O Post-operative delirium is associated with poor cognitive outcome and dementia Dement Geriatr Cogn Disord 2006 21 221 227 10.1159/000091022 16428883

[B22] Fick DM Foreman M Consequences of not recognizing delirium superimposed on dementia in hospitalized elderly individuals J Gerontol Nurs 2000 26 30 40 10776167 10.3928/0098-9134-20000101-09

[B23] Voyer P McCusker J Cole M Khomenko L Influence of prior cognitive impairment on the severity of delirium symptoms among older patients J Neurosci Nurs 2006 38 90 101 10.1097/01376517-200604000-00004 16681289

[B24] Sabbagh M Lahti T Connor D Caviness J Shill H Vedders L Mahant P Samanta J Burns R Evidente V Functional ability correlates with cognitive impairment in Parkinson's disease and Alzheimer's disease Dement Geriatr Cogn Disord 2007 24 327 334 10.1159/000108340 17851237

[B25] McGuire L Ford E Ajani U Cognitive functioning as a predictor of functional disability in later life American Journal of Geriatric Psych 2006 14 36 42 10.1097/01.JGP.0000192502.10692.d6 16407580

[B26] Njegovan V Man-Son-Hing M Mitchell S Molnar F The hierarchy of functional loss associated with cognitive decline in older persons The Journals of Gerontology Series A: Biological Sciences and Medical Sciences 2001 56 M638 643 10.1093/gerona/56.10.m638 11584037

[B27] Perry RJ Hodges JR Attention and executive deficits in Alzheimer's disease. A critical review Brain 1999 122 Pt 3 383 404 10094249 10.1093/brain/122.3.383

[B28] Sheridan PL Solomont J Kowall N Hausdorff JM Influence of executive function on locomotor function: Divided attention increases gait variability in Alzheimer's disease J Am Geriatr Soc 2003 51 1633 1637 10.1046/j.1532-5415.2003.51516.x 14687395

[B29] Parasuraman R Morris RG, Becker JT Attentional functioning in Alzheimer's disease Cognitive neuropsychology of Alzheimer's disease 2004 Oxford: Oxford University Press 81 10221493755

[B30] Chiu YC Algase D Whall A Liang J Liu HC Lin KN Wang PN Getting lost: directed attention and executive functions in early Alzheimer's disease patients Dement Geriatr Cogn Disord 2004 17 174 180 10.1159/000076353 14739541

[B31] Voyer P Richard S Doucet L Carmichael P Predisposing factors associated with delirium among demented long-term care residents Clin Nurs Res 2009 18 153 171 10.1177/1054773809333434 19377042

[B32] Lerner A Hedera P Koss E Stuckey J Friedland R Delirium in Alzheimer disease Alzheimer Dis Assoc Disord 1997 11 16 20 10.1097/00002093-199703000-00004 9071440

[B33] Fick D Kolanowski A Waller J Inouye S Delirium superimposed on dementia in a community-living managed care population: A three year retrospective study of prevalence, costs, and utilization Journals of Gerontology: Medical Sciences, 60A 2005 6 748 753 10.1093/gerona/60.6.748 15983178

[B34] Hajjar E Hanlon J Sloane R Lindblad C Pieper C Ruby C Branch L Schmader K Unnecessary drug use in frail older people at hospital discharge J Am Geriatr Soc 2005 53 1518 1523 10.1111/j.1532-5415.2005.53523.x 16137281

[B35] Meador K Cognitive side effects of medications Neurol Clin 1998 16 141 155 10.1016/S0733-8619(05)70371-6 9421545

[B36] Fick D Kolanowski A Waller J High prevalence of central nervous system medications in community-dwelling older adults with dementia over a three-year period Aging Ment Health 2007 11 588 595 10.1080/13607860601086629 17882597

[B37] Tune L Egeli S Acetylcholine and delirium Dement Geriatr Cogn Disord 2000 10 342 344 10.1159/000017167 10473936

[B38] van Munster B Korevaar J de Rooij S Levi M Zwinderman A The association between delirium and the apolipoprotein E [varepsilon] 4 allele in the elderly Psychiatr Genet 2007 17 261 10.1097/YPG.0b013e3280c8efd4 17728664

[B39] Leung J Sands L Wang Y Poon A Kwok P Kane J Pullinger C Apolipoprotein E e4 allele increases the risk of early postoperative delirium in older patients undergoing noncardiac surgery Anesthesiology 2007 107 406 411 10.1097/01.anes.0000278905.07899.df 17721242

[B40] Adamis D Treloar A Martin F Gregson N Hamilton G Macdonald A APOE and cytokines as biological markers for recovery of prevalent delirium in elderly medical inpatients Int J Geriatr Psychiatry 2007 22 688 694 10.1002/gps.1732 17203511

[B41] Ely E Girard T Shintani A Jackson J Gordon S Thomason J Pun B Canonico A Light R Pandharipande P Apolipoprotein E4 polymorphism as a genetic predisposition to delirium in critically ill patients* Crit Care Med 2007 35 112 117 10.1097/01.CCM.0000251925.18961.CA 17133176

[B42] Kempermann G The neurogenic reserve hypothesis: what is adult hippocampal neurogenesis good for? Trends Neurosci 2008 31 163 169 10.1016/j.tins.2008.01.002 18329110

[B43] Carnes M Howell T Rosenberg M Francis J Hildebrand C Knuppel J Physicians vary in approaches to the clinical management of delirium J Am Geriatr Soc 2003 51 234 239 10.1046/j.1532-5415.2003.51063.x 12558721

[B44] Pitkälä K Laurila J Strandberg T Tilvis R Multicomponent geriatric intervention for elderly inpatients with delirium: a randomized, controlled trial The Journals of Gerontology Series A: Biological Sciences and Medical Sciences 2006 61 176 10.1093/gerona/61.2.176 16510862

[B45] Cole M Primeau F Bailey R Bonnycastle M Masciarelli F Engelsmann F Pepin M Ducic D Systematic intervention for elderly inpatients with delirium: a randomized trial Can Med Assoc J 1994 151 965 970 PMC1337283 7922932

[B46] Cole M McCusker J Bellavance F Primeau F Bailey R Bonnycastle M Laplante J Systematic detection and multidisciplinary care of delirium in older medical inpatients: a randomized trial Can Med Assoc J 2002 167 753 759 PMC126506 12389836

[B47] Ellul J Archer N Foy C Poppe M Boothby H Nicholas H Brown R Lovestone S The effects of commonly prescribed drugs in patients with Alzheimer's disease on the rate of deterioration J Neurol Neurosurg Psychiatry 2007 78 233 239 17012333 10.1136/jnnp.2006.104034 PMC2117629

[B48] McShane R Keene J Gedling K Fairburn C Jacoby R Hope T Do neuroleptic drugs hasten cognitive decline in dementia? Prospective study with necropsy follow up BMJ 1997 314 266 270 9022490 10.1136/bmj.314.7076.266 PMC2125727

[B49] Stern Y What is cognitive reserve? Theory and research application of the reserve concept J Int Neuropsychol Soc 2002 8 448 460 11939702

[B50] Perneczky R Haussermann P Drzezga A Boecker H Granert O Feurer R Forstl H Kurz A Fluoro-deoxy-glucose positron emission tomography correlates of impaired activities of daily living in dementia with Lewy bodies: Implications for cognitive reserve American Journal of Geriatric Psych 2009 17 188 195 10.1097/JGP.0b013e3181961a6f 19454846

[B51] van Praag H Kempermann G Gage F Neural consequences of enviromental enrichment Nature Reviews Neuroscience 2000 1 191 198 11257907 10.1038/35044558

[B52] Valenzuela MJ Sachdev P Brain reserve and dementia: a systematic review Psychol Med 2006 36 441 454 16207391 10.1017/S0033291705006264

[B53] Jones RN Yang FM Zhang Y Kiely DK Marcantonio ER Inouye SK Does educational attainment contribute to risk for delirium? A potential role for cognitive reserve J Gerontol A Biol Sci Med Sci 2006 61 1307 1311 17234825 10.1093/gerona/61.12.1307

[B54] Valenzuela MJ Sachdev P Assessment of complex mental activity across the lifespan: development of the Lifetime of Experiences Questionnaire (LEQ) Psychol Med 2007 37 1015 1025 10.1017/S003329170600938X 17112402

[B55] Inouye S Ferrucci L Elucidating the pathophysiology of delirium and the interrelationship of delirium and dementia The journals of gerontology Series A, Biological sciences and medical sciences 2006 61 1277 1280 17234820 10.1093/gerona/61.12.1277 PMC2645654

[B56] Stern Y Cognitive reserve and Alzheimer disease Alzheimer Dis Assoc Disord 2006 20 112 117 10.1097/01.wad.0000213815.20177.19 16772747

[B57] Valenzuela M Sachdev P Rundek T Bennett D Cognitive leisure activities, but not watching TV, for future brain benefits Neurology 2006 67 729 10.1212/01.wnl.0000239615.49557.63 16924044

[B58] Reyes-Ortiz CA Delirium, dementia and brain reserve J Am Geriatr Soc 1997 45 778 779 9180681 10.1111/j.1532-5415.1997.tb01492.x

[B59] Murray A Levkoff S Wetle T Beckett L Cleary P Schor J Lipsitz L Rowe J Evans D Acute delirium and functional decline in the hospitalized elderly patient J Gerontol 1993 48 M181 186 8366260 10.1093/geronj/48.5.m181

[B60] Ball K Berch D Helmers K Jobe J Leveck M Marsiske M Morris J Rebok G Smith D Tennstedt S Effects of cognitive training interventions with older adults: a randomized controlled trial JAMA 2002 288 2271 2281 10.1001/jama.288.18.2271 12425704 PMC2916176

[B61] Willis S Tennstedt S Marsiske M Ball K Elias J Koepke K Morris J Rebok G Unverzagt F Stoddard A Long-term effects of cognitive training on everyday functional outcomes in older adults JAMA 2006 296 2805 2814 10.1001/jama.296.23.2805 17179457 PMC2910591

[B62] Spector A Thorgrimsen L Woods B Royan L Davies S Butterworth M Orrell M Efficacy of an evidence-based cognitive stimulation therapy programme for people with dementia: randomised controlled trial Br J Psychiatry 2003 183 248 254 10.1192/bjp.183.3.248 12948999

[B63] Boron J Willis S Schaie K Cognitive training gain as a predictor of mental status The Journals of Gerontology Series B: Psychological Sciences and Social Sciences 2007 62 P45 52 10.1093/geronb/62.1.p45 17284557

[B64] Vance D Webb N Marceaux J Viamonte S Foote A Ball K Mental stimulation, neural plasticity, and aging: directions for nursing research and practice J Neurosci Nurs 2008 40 241 249 10.1097/01376517-200808000-00008 18727340

[B65] Clare L Woods R Cognitive training and cognitive rehabilitation for people with early-stage Alzheimer s disease: A review Neuropsychological Rehabilitation 2004 14 385 401 10.1080/09602010443000074

[B66] Clare L Woods R Moniz C Orrell M Spector A Cognitive rehabilitation and cognitive training for early-stage Alzheimer's disease and vascular dementia Cochrane database of systematic reviews 2003 4 CD003260 10.1002/14651858.CD003260 14583963

[B67] De Vreese L Neri M Fioravanti M Belloi L Zanetti O Memory rehabilitation in Alzheimer's disease: a review of progress Int J Geriatr Psychiatry 2001 16 794 809 10.1002/gps.428 11536347

[B68] Loewenstein D Acevedo A Czaja S Duara R Cognitive rehabilitation of mildly impaired Alzheimer disease patients on cholinesterase inhibitors American Journal of Geriatric Psych 2004 12 395 402 10.1176/appi.ajgp.12.4.395 15249277

[B69] Olazaran J Muniz R Reisberg B Pena-Casanova J Del Ser T Cruz-Jentoft A Serrano P Navarro E Garcia de La Rocha M Frank A Benefits of cognitive-motor intervention in MCI and mild to moderate Alzheimer disease Neurology 2004 63 2348 2353 15623698 10.1212/01.wnl.0000147478.03911.28

[B70] Wenisch E Cantegreil-Kallen I De Rotrou J Garrigue P Moulin F Batouche F Richard A DE S Cognitive stimulation intervention for elders with mild cognitive impairment compared with normal aged subjects: preliminary results Aging Clinical and Experimental Research 2007 19 316 322 17726363 10.1007/BF03324708

[B71] Sitzer D Twamley E Jeste D Cognitive training in Alzheimer's disease: a meta analysis of the literature Acta Psychiatr Scand 2006 114 75 90 10.1111/j.1600-0447.2006.00789.x 16836595

[B72] Requena C López Ibor M Maestú F Campo P López Ibor J Ortiz T Effects of cholinergic drugs and cognitive training on dementia Dement Geriatr Cogn Disord 2004 18 50 54 10.1159/000077735 15084794

[B73] Requena C Maestu F Campo P Fernandez A Ortiz T Effects of cholinergic drugs and cognitive training on dementia: 2-year follow-up Dement Geriatr Cogn Disord 2006 22 339 345 10.1159/000095600 16954689

[B74] Spector A Woods B Orrell M Cognitive stimulation for the treatment of Alzheimer's disease Expert Review of Neurotherapeutics 2008 8 751 757 10.1586/14737175.8.5.751 18457532

[B75] Kolanowski A Buettner L Fick D Fitzsimmons S Cornacchione M Instituting cognitive rehabilitation in post-acute care Annals of Long-Term Care 2008 16 40 46

[B76] Kolanowski A Fick D Clare L Steis M Boustani M Litaker M Pilot study of a nonpharmacological intervention for delirium superimposed on dementia Research in Gerontological Nursing 2010 in press 10.3928/19404921-20101001-98 21053841

[B77] Kolanowski A Litaker M Buettner L Efficacy of theory-based activities for behavioral symptoms of dementia Nurs Res 2005 54 219 228 16027564 10.1097/00006199-200507000-00003

[B78] Kolanowski A Litaker M Buettner L Moeller J Costa P A randomized clinical trial of theory-based activities for the behavioral symptoms of dementia in nursing home residents J Am Geriatr Soc in press 10.1111/j.1532-5415.2011.03449.x PMC3381903 21649633

[B79] Choi J Medalia A Factors associated with a positive response to cognitive remediation in a community psychiatric sample Psychiatr Serv 2005 56 602 604 10.1176/appi.ps.56.5.602 15872171

[B80] Voyer P Cole M McCusker J Belzile É Prevalence and symptoms of delirium superimposed on dementia Clin Nurs Res 2006 15 46 66 10.1177/1054773805282299 16410622

[B81] Siddiqi N Stockdale R Britton AM Holmes J Interventions for preventing delirium in hospitalised patients Cochrane Database Syst Rev 2007 2 CD005563 17443600 10.1002/14651858.CD005563.pub2

[B82] Blessed G Tomlinson B Roth M The association between quantitative measures of dementia and of senile change in the cerebral grey matter of elderly subjects The British Journal of Psychiatry 1968 114 797 811 10.1192/bjp.114.512.797 5662937

[B83] Hughes C Berg L Danziger W Coben L Martin R A new clinical scale for the staging of dementia The British Journal of Psychiatry 1982 140 566 572 10.1192/bjp.140.6.566 7104545

[B84] Buckwalter K Grey M Bowers B McCarthy A Gross D Funk M Beck C Intervention research in highly unstable environments Res Nurs Health 2009 32 110 121 10.1002/nur.20309 19035619 PMC2923040

[B85] Rantz M Hicks L Petroski G Madsen R Mehr D Conn V Zwygart-Staffacher M Maas M Stability and sensitivity of nursing home quality indicators The Journals of Gerontology Series A: Biological Sciences and Medical Sciences 2004 59 79 82 10.1093/gerona/59.1.m79 14718490

[B86] Buettner L Kolanowski A Yu F Recreational games: Simple and effective cognitive stimulation programs for residents with dementia in long-term settings American Journal of Recreation Therapy 2007 6 25 30

[B87] Spector A Thorgrimsen L Woods R Orrell M Making a Difference: An Evidence-based Group Programme to Offer Cognitive Stimulation Therapy (CST) to People with Dementia 2006 London: Hawker Publications

[B88] Buettner L Martin S Therapeutic recreation in the nursing home 1995 State College, PA: Venture Pub

[B89] Fitzsimmons S Brain fitness 2008 State College, PA: Venture Pub

[B90] Goldberg E The new executive brain: frontal lobes in a complex world 2009 New York: Oxford University Press

[B91] Valenzuela M Sachdev P Can cognitive exercise prevent the onset of dementia? Systematic review of randomized clinical trials with longitudinal follow-up American Journal of Geriatric Psych 2009 17 179 187 10.1097/JGP.0b013e3181953b57 19225276

[B92] Engelman K Mathews R Altus D Restoring dressing independence in persons with Alzheimer's disease: A pilot study American Journal of Alzheimer's Disease and Other Dementias 2002 17 37 43 10.1177/153331750201700102 11831419 PMC10833979

[B93] Green C Bavelier D Exercising your brain: A review of human brain plasticity and training-induced learning Psychol Aging 2008 23 692 701 19140641 10.1037/a0014345 PMC2896818

[B94] Riddick C Keller J Coyle C, Kinney W, Riley B, Shanks J The benefits of therapeutic recreation in gerontology Benefits of Therapeutic Recreation: A Consensus View 1991 Philadelphia: Temple University, Therapeutic Recreation Program 151 204

[B95] Buettner L Simple Pleasures: A multilevel sensorimotor intervention for nursing home residents with dementia American Journal of Alzheimer's Disease and Other Dementias 1999 14 41 52 10.1177/15333175990140010321522051

[B96] Buettner L Fitzsimmons S Introduction to evidence-based recreational therapy Annual in Therapeutic Recreation 2007 15 12 90

[B97] Buettner L Kolanowski A Practice guidelines for recreation therapy in the care of people with dementia Geriatr Nur 2003 24 18 23 10.1067/mgn.2003.19 12598862

[B98] Kolanowski A Buettner L Moeller J Treatment fidelity plan for an activity intervention designed for persons with dementia American Journal of Alzheimer's Disease and Other Dementias 2006 21 326 332 10.1177/1533317506291074 17062551 PMC10832628

[B99] Bellg A Borrelli B Resnick B Hecht J Minicucci D Ory M Ogedegbe G Orwig D Ernst D Czajkowski S Enhancing treatment fidelity in health behavior change studies: Best practices and recommendations from the NIH Behavior Change Consortium Health Psychol 2004 23 443 451 15367063 10.1037/0278-6133.23.5.443

[B100] Morandi A Solberg L Habermann R Cleeton P Peterson E Ely E Schnelle J Documentation and management of words associated with delirium among elderly patients in postacute care: a pilot investigation Journal of the American Medical Directors Association 2009 10 330 334 10.1016/j.jamda.2009.02.002 19497545

[B101] Fick D Hodo D Lawrence F Inouye S Recognizing delirium superimposed on dementia: Assessing nurses' knowledge using case vignettes J Gerontol Nurs 2007 33 40 47 17310662 10.3928/00989134-20070201-09 PMC2247368

[B102] Allison J Wall T Spettell C Calhoun J Fargason C Kobylinski R Farmer R Kiefe C The art and science of chart review Jt Comm J Qual Improv 2000 26 115 136 10709146 10.1016/s1070-3241(00)26009-4

[B103] Cassidy L Marsh G Holleran M Ruhl L Methodology to improve data quality from chart review in the managed care setting Am J Manag Care 2002 8 787 793 12234019

[B104] Freeman B Smith N Curtis C Huckett L Mill J Craig I DNA from buccal swabs recruited by mail: evaluation of storage effects on long-term stability and suitability for multiplex polymerase chain reaction genotyping Behav Genet 2003 33 67 72 10.1023/A:1021055617738 12645823

[B105] Ertekin-Taner N Genetics of Alzheimer's disease: a centennial review Neurol Clin 2007 25 611 667 10.1016/j.ncl.2007.03.009 17659183 PMC2735049

[B106] Nasreddine Z Phillips N Bédirian V Charbonneau S Whitehead V Collin I Cummings J Chertkow H The Montreal Cognitive Assessment, MoCA: a brief screening tool for mild cognitive impairment J Am Geriatr Soc 2005 53 695 699 10.1111/j.1532-5415.2005.53221.x 15817019

[B107] Inouye S Van Dyck C Alessi C Balkin S Siegal A Horwitz R Clarifying confusion: the confusion assessment method. A new method for detection of delirium Ann Intern Med 1990 113 941 948 2240918 10.7326/0003-4819-113-12-941

[B108] Voyer P Richard S Doucet L Carmichael P Detecting delirium and subsyndromal delirium using different diagnostic criteria among demented long-term care residents Journal of the American Medical Directors Association 2009 10 181 188 10.1016/j.jamda.2008.09.006 19233058

[B109] Pompei P Foreman M Cassel C Alessi C Cox D Detecting delirium among hospitalized older patients Arch Intern Med 1995 155 301 307 10.1001/archinte.155.3.301 7832602

[B110] Han L McCusker J Cole M Abrahamowicz M Primeau F Elie M Use of medications with anticholinergic effect predicts clinical severity of delirium symptoms in older medical inpatients Arch Intern Med 2001 161 1099 1105 10.1001/archinte.161.8.1099 11322844

[B111] Yang F Marcantonio E Inouye S Kiely D Rudolph J Fearing M Jones R Phenomenological subtypes of delirium in older persons: patterns, prevalence, and prognosis Psychosomatics 2009 50 248 254 10.1176/appi.psy.50.3.248 19567764 PMC2705885

[B112] Trzepacz P Baker R Greenhouse J A symptom rating scale for delirium Psychiatry Res 1988 23 89 97 10.1016/0165-1781(88)90037-6 3363018

[B113] Wechsler D Wechsler Adult Intelligence Scales - Revised (WAIS-R) 1981 New York: Psychological Corporation

[B114] Ramsay M Reynolds C Separate digits tests: A brief history, a literature review, and a reexamination of the factor structure of the Test of Memory and Learning (TOMAL) Neuropsychol Rev 1995 5 151 171 10.1007/BF02214760 8653107

[B115] Palmer RM Meldon SW Hazzard WR Digit span test in acute care Principles of Geriatric Medicine and Gerontology 2003 5 New York: McGraw-Hill Professional 157 168

[B116] Strauss E Sherman E Spreen O A Compendium of Neuropsychological Tests: Administration, Norms, and Commentary 2006 3 Oxford; New York: Oxford University Press

[B117] Royall DR Cordes JA Polk M CLOX: An executive clock drawing task J Neurol Neurosurg Psychiatry 1998 64 588 594 10.1136/jnnp.64.5.588 9598672 PMC2170069

[B118] Sainsbury A Seebass G Bansal A Young J Reliability of the Barthel Index when used with older people Age Ageing 2005 34 228 232 10.1093/ageing/afi063 15863408

[B119] Mahoney F Barthel D Functional evaluation: the Barthel Index Md State Med J 1965 14 61 65 14258950

[B120] Shah S Vanclay F Cooper B Improving the sensitivity of the Barthel Index for stroke rehabilitation J Clin Epidemiol 1989 42 703 709 10.1016/0895-4356(89)90065-6 2760661

[B121] van Doorn C Bogardus S Williams C Concato J Towle V Inouye S Risk adjustment for older hospitalized persons: A comparison of two methods of data collection for the Charlson Index J Clin Epidemiol 2001 54 694 701 10.1016/S0895-4356(00)00367-X 11438410

[B122] Warden V Hurley AC Volicer L Development and psychometric evaluation of the pain assessment in advanced dementia (PAINAD) scale Journal of the American Medical Directors Association 2003 4 9 15 10.1016/S1525-8610(04)70258-3 12807591

[B123] Herr K Bjoro K Decker S Tools for assessment of pain in nonverbal older adults with dementia: a state-of-the-science review J Pain Symptom Manage 2006 31 170 192 10.1016/j.jpainsymman.2005.07.001 16488350

[B124] Boustani M Campbell N Munger S Maidment I Fox C Impact of anticholinergics on the aging brain: a review and practical application Aging Health 2008 4 311 320 10.2217/1745509X.4.3.311

[B125] Campbell N Boustani M Limbil T Ott C Fox C Maidment I Schubert C Munger S Fick D Miller D The cognitive impact of anticholinergics: A clinical review Clinical Interventions in Aging 2009 4 225 233 19554093 10.2147/cia.s5358 PMC2697587

[B126] Kovach C Magliocco J Late-stage dementia and participation in therapeutic activities Appl Nurs Res 1998 11 167 173 10.1016/S0897-1897(98)80285-1 9852659

[B127] Marcantonio E Michaels M Resnick N Diagnosing delirium by telephone J Gen Intern Med 1998 13 621 623 10.1046/j.1525-1497.1998.00185.x 9754518 PMC1497007

[B128] Baron R Kenny D The moderator-mediator variable distinction in social psychological research: Conceptual, strategic, and statistical considerations J Pers Soc Psychol 1986 51 1173 1182 3806354 10.1037//0022-3514.51.6.1173

[B129] Judd CM Kenny DA McClelland GH Estimating and testing mediation and moderation in within-subject designs Psychological Methods 2001 6 115 134 11411437 10.1037/1082-989x.6.2.115

[B130] MacKinnon D Lockwood C Hoffman J West S Sheets V A comparison of methods to test mediation and other intervening variable effects Psychological Methods 2002 7 83 104 11928892 10.1037/1082-989x.7.1.83 PMC2819363

